# Infective Pseudoaneurysm of the Anterior Mitral Leaflet Accompanied by Aortic Wall Vegetation 

**Published:** 2016-01-13

**Authors:** Ali Hosseinsabet

**Affiliations:** Tehran Heart Center, Tehran University of Medical Sciences, Tehran, Iran.

**Keywords:** *Endocarditis*, *Aneurysm, false*, *Mitral valve*, *Aorta*

 A 46-year-old man with a history of cigarette smoking was referred to our hospital for further evaluation of infective endocarditis. He had a history of fever of 45 days’ duration. Previously in workup for fever, the patient had undergone transthoracic echocardiography, showing vegetations on the aortic and mitral valves, hence his referral to our hospital. On physical examination, he was febrile with no peripheral signs of endocarditis. On cardiac auscultation, a systolic murmur in the apex and a diastolic murmur in the left parasternal border could be heard. Electrocardiography showed sinus tachycardia with poor R-wave progression in V_1_-V_4_. Blood cultures were negative probably due to the previous administration of antibiotics. Transthoracic and transesophageal echocardiographic examinations revealed vegetations on the anterior mitral leaflet with pseudoaneurysm formation, which had ruptured to the left ventricle, and moderate to severe mitral regurgitation (Figure 1, Videos 1 & 2). Also, these imaging studies showed destructive aortic valve and large vegetations with severe aortic regurgitation and a highly mobile vegetation on the anterior wall of the aorta, near the right coronary artery (RCA) ostium prolapsing to the proximity of the RCA ostium (Figure 2, Videos 3 & 4). There was no turbulent flow in the ostium of the RCA in color flow Doppler study. The left ventricle was severely enlarged with moderate systolic dysfunction and global hypokinesia. Coronary computed tomography angiography was unremarkable. Finally, mitral and aortic valve replacement was done for the patient, and postoperative antibiotics were continued for 4 weeks. The patient had an uneventful recovery period.

Infective endocarditis can be seen in daily clinical practice, but there are some unusual cases that require profound attention. Highly mobile vegetations on the aortic wall prolapsing to the proximity of the coronary artery ostium is one of these unusual cases. Accordingly, meticulous evaluation of all cardiac structures in patients with infective endocarditis is necessary.

**Figure 1:**
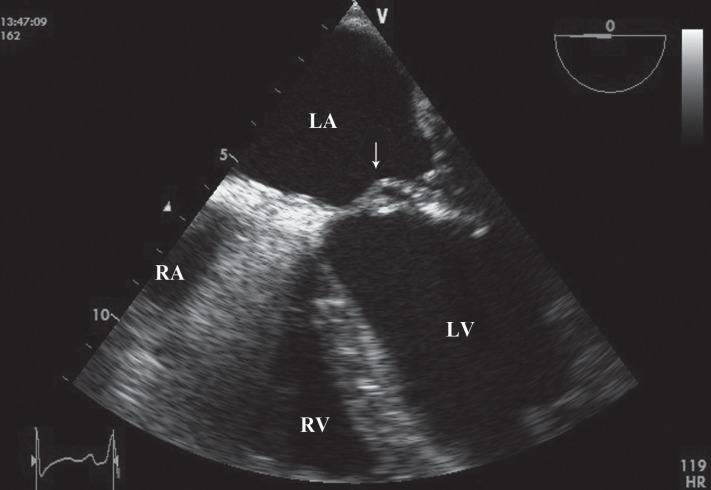
Pseudoaneurysm of the anterior mitral leaflet on transesophageal echocardiography (arrow)

LA, Left atrium; LV, Left ventricle; RA, Right atrium; RV, Right ventricle

**Figure 2:**
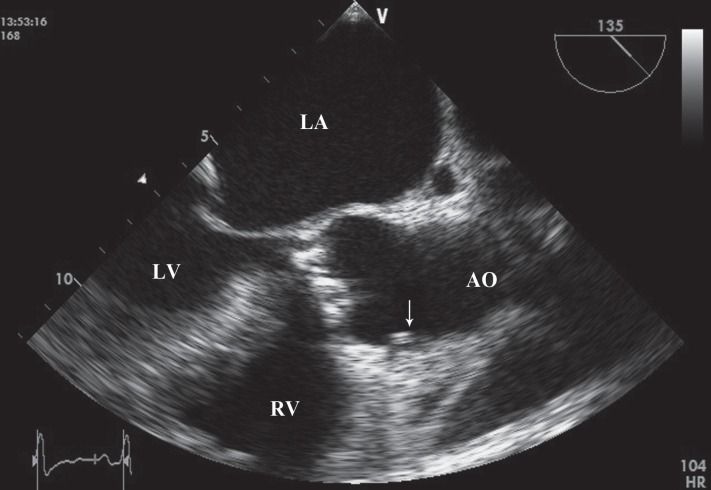
Vegetation on the anterior aortic wall prolapsing to the proximity of the right coronary ostium on transesophageal echocardiography (arrow)

AO, Aorta; LA, Left atrium; LV, Left ventricle; RV, Right ventricle

***To watch the following videos, please refer to the relevant URLs:***

Video 1. Pseudoaneurysm of the anterior mitral leaflet on transesophageal echocardiography in 2D.

http://jthc.tums.ac.ir/index.php/jthc/article/view/518/423

Video 2. Pseudoaneurysm of the anterior mitral leaflet on transesophageal echocardiography in color flow Doppler study.

http://jthc.tums.ac.ir/index.php/jthc/article/view/518/424

Video 3. Vegetation on the anterior aortic wall prolapsing to the proximity of the right coronary ostium on transesophageal echocardiography (long-axis view).

http://jthc.tums.ac.ir/index.php/jthc/article/view/518/425

Video 4. Vegetation on the anterior aortic wall prolapsing to the proximity of the right coronary ostium on transesophageal echocardiography (short-axis view).

http://jthc.tums.ac.ir/index.php/jthc/article/view/518/426

## Notes:

***This paper should be cited as: ***Hosseinsabet A. Infective Pseudoaneurysm of the Anterior Mitral Leaflet Accompanied by Aortic Wall Vegetation. J The Univ Heart Ctr 2015;11(1):46-47.

